# Antiviral Agents: Discovery to Resistance

**DOI:** 10.3390/v12040406

**Published:** 2020-04-07

**Authors:** Catherine S. Adamson

**Affiliations:** School of Biology, Biomedical Sciences Research Complex, University of St Andrews, St Andrews KY16 9ST, Scotland, UK; csa21@st-andrews.ac.uk

In the midst of the SARS-CoV-2/Covid-19 outbreak the need for research into, and development of, antiviral agents is brought into sharp focus worldwide for scientists, governments and the public alike. This special issue includes primary research into new antiviral agents with activity against a range of clinically and economically important viruses. New antiviral strategies are discussed in six review articles.

SARS-CoV-2 is a newly emerged β-Coronavirus with sustained human-to-human transmission via the respiratory tract. Influenza viruses are another group of respiratory viruses, which account for 300–600 K deaths annually and represent an ever-present pandemic threat. Emergence of drug-resistant influenza strains is driving research into new anti-influenza drug candidates with different modes of action. In this issue, two papers report compounds with anti-influenza activity against a range of influenza A viruses that inhibit early steps in influenza replication not targeted by clinically approved anti-influenza drugs [1,2]. A further study reports that respiratory syncytial virus is inhibited in vitro and in vivo by natural product plant extracts in which phenolic glycoside acetoside was the active chemical component [3]. 

Drug resistance is an ongoing issue in the global rollout of combination antiretroviral therapy for human immunodeficiency virus (HIV) prevention and treatment. Tadesse et al. report the prevalence of pretreatment drug resistance among HIV-infected children in the resource-limited setting of Ethiopia [4]. Lectins are experimental HIV-1 entry inhibitors. Shahid et al. engineered the lectin microvirin to contain two carbohydrate-binding domains, this reduced its anti-HIV potency but anti-hepatitis C virus (HCV) activity was demonstrated [5]. Sherpa and Le Grice review recent research into the structural fluidity of the HIV Rev response element (RRE) and its potential as a target for therapeutic intervention [6].

Antiviral agents are not available against many emerging and neglected viruses. This issue features four research papers reporting new antiviral compound candidates against zika virus [7,8], hantaan virus [9] and herpes B virus [10]. Measles virus is considered a re-emerging virus due to recent decline in vaccine coverage. Ferren et al. provide a comprehensive review on measles virus with a focus on central nervous system complications and the most advanced therapeutic approaches [11].

In addition to study into the zoonotic herpes B virus, this issue also includes two primary research papers investigating antiviral approaches targeting reactivation of latent herpesvirus infection. Anderson et al. demonstrated that the antipsychotic drug cloazapine inhibits epstein-barr virus (EBV) lytic reactivation [12]. In contrast, Chen et al. explore the potential application of genome-editing transcription activator-like effector nucleases (TALENs) against latent murine cytomegalovirus (CMV) infections [13]. The special issue also includes a review of the current antiviral strategies against human CMV [14] and a more focused review by Adamson and Nevels, which argues the case for the development of novel anti-CMV compounds by targeting major immediate-early (IE) gene expression or protein function [15]. RNA viruses can also establish persistent infections and this issue includes a study investigating the acquisition of drug resistance to fluoxetine during treatment of pancreatic cell-lines persistently infected with coxsackievirus B4 [16].

Research into antiviral agents against economically important animal viruses is also included in this issue, with three primary papers reporting antiviral agents with inhibitory activity against porcine circovirus type 2 (PCV2) [17] and porcine reproductive and respiratory syndrome virus (PRRSV) [18,19]. The study by Yu et al. demonstrates that antiviral effects of Ginsenoside Rg1 against PRRSV can be attributed to reductions in levels of host cell pro-inflammatory cytokines and suppression of NF-κΒ signalling [18].

As mentioned, this special issue includes reviews into antiviral strategies against HIV, measles and CMV [6,11,14,15]. In addition, the issue contains reviews that discuss sphingolipids as potential therapeutic targets against enveloped human RNA viruses [20] and human antimicrobial peptides as therapeutics for viral infections [21].

Finally, I would like to thank all the authors, reviewers and editors, who made this issue possible, both at Viruses and in the wider academic community.
